# Reducing DNS Traffic to Enhance Home IoT Device Privacy

**DOI:** 10.3390/s24092690

**Published:** 2024-04-24

**Authors:** Marta Moure-Garrido, Carlos Garcia-Rubio, Celeste Campo

**Affiliations:** Department of Telematic Engineering, University Carlos III of Madrid, Av. de la Universidad 30, E-28911 Leganes, Spain; mamoureg@it.uc3m.es (M.M.-G.); celeste@it.uc3m.es (C.C.)

**Keywords:** DNS, IoT privacy, network traffic

## Abstract

The deployment of Internet of Things (IoT) devices is widespread in different environments, including homes. Although security is incorporated, homes can become targets for cyberattacks because of their vulnerabilities. IoT devices generate Domain Name Server (DNS) traffic primarily for communication with Internet servers. In this paper, we present a detailed analysis of DNS traffic from IoT devices. The queried domains are highly distinctive, enabling attackers to easily identify the IoT device. In addition, we observed an unexpectedly high volume of queries. The analysis reveals that the same domains are repeatedly queried, DNS queries are transmitted in plain text over User Datagram Protocol (UDP) port 53 (Do53), and the excessive generation of traffic poses a security risk by amplifying an attacker’s ability to identify IoT devices and execute more precise, targeted attacks, consequently escalating the potential compromise of the entire IoT ecosystem. We propose a simple measure that can be taken to reduce DNS traffic generated by IoT devices, thus preventing it from being used as a vector to identify the types of devices present in the network. This measure is based on the implementation of the DNS cache in the devices; caching few resources increases privacy considerably.

## 1. Introduction

The Internet of Things (IoT) is a term that refers to the interconnection of physical objects through the Internet. These objects range from thermostats, security cameras, and household appliances to lighting or the presence sensors, among others. The home is one of the areas where IoT is having a significant impact. IoT devices connected to homes, known as smart homes, offer a wide variety of advantages, such as convenience, security, and energy efficiency. The deployment of IoT is growing at an exponential rate. According to some studies, the number of connected IoT devices is expected to reach one trillion by 2030 [1]. This growth is attributed to the reduction in the costs of IoT devices and the increasing demand for applications.

Connected IoT devices in homes can be vulnerable to cyber attacks, especially if the user does not configure and update them properly. These attacks begin by identifying the type of device, manufacturer, and model and then exploiting any known vulnerabilities. In [2], it is shown how a third party could indirectly determine the type of product, manufacturer, and model of devices in our home, exploiting the unencrypted information in the response data of the application layer of IoT devices. This information can be inferred using specific tools such as ZMap [3] and comparing messages with databases, and even using machine learning techniques to identify home devices [4]. Once the device type is identified, an attacker can exploit known weaknesses or simply use factory preconfigured keys (not changed by most users) to access devices for (i) either obtaining information violating their privacy or (ii) use in distributed denial-of-service (DDoS) attacks [5].

Fortunately, most devices nowadays use secure versions of application-level protocols (such as CoAP, MQTT, or HTTP), preventing someone from observing the traffic and obtaining information through the method explained earlier. However, Domain Name Server (DNS) queries often continue to be transmitted in plaintext and can be used to extract information from IoT devices present on the network [6].

The aim of this paper is to study the DNS traffic generated by a wide range of devices found in homes, most of which are IoT devices but also including computers or mobile phones. We will examine the different characteristics of queries based on the type of device and apply different machine learning techniques to identify different devices based on DNS features extracted from network traffic. The main goal is to analyze the impact of the queries of transmitted DNS traffic on the identification of IoT devices connected to the network.

We propose some simple measures that can be taken to reduce DNS traffic generated by IoT devices, thus preventing it from being used as a vector to identify the types of devices present in the network. This study evaluates the proposed measures by analyzing the impact of DNS traffic on the identification of IoT devices. The performed evaluation reveals that the proposed measures reduce the generated DNS traffic, restricting the sensitive information of a device that would be at risk of being exposed to an attack.

This paper proposes an improvement to the privacy of IoT devices by reducing excessive DNS traffic. The key contributions of this paper are as follows: (i) privacy analysis of IoT devices in a home network focusing on DNS traffic; (ii) proposal to reduce the DNS traffic exposed to possible attacks based on the implementation of the DNS cache in the devices; (iii) study of the appropriate cache size of an IoT device to reduce the device information revealed in DNS queries; and (iv) evaluation of the proposed solution through simulations.

The paper is organized as follows: Section 2 presents the related work on IoT device identification. The DNS traffic analysis relying on domain names queried is provided in Section 3, while Section 4 provides a more detailed analysis of the DNS traffic behavior pattern to provide insights into the presence of the DNS protocol in IoT devices. We examine the high volume of DNS queries performed by IoT devices in Section 5. Section 6 gives the implementation of a simple measure proposed to reduce DNS traffic and the comprehensive evaluation performed. Finally, conclusions are offered in Section 7.

## 2. Related Work

In recent years, there has been a surge of research efforts aimed at identifying IoT devices through their network traffic patterns. This ability to identify IoT devices within a network is crucial for assessing the potential vulnerability to cybersecurity attacks that exploit these devices as attack vectors. A prime example of such an attack is the Mirai botnet [7], which leveraged a simple vulnerability in specific IoT devices to infect over 60,000 devices.

Attacks are becoming increasingly sophisticated, and it is difficult to detect and mitigate the threats present in the IoT ecosystem. However, the improvement in security and detection of this type of attack is feasible by applying different techniques such as machine learning [8].

Feature extraction from network traffic is critical in the identification of IoT devices. An evaluation of feature extraction is presented in [4]. Features can be at the packet level or at the flow level. The most important flow-level features can be categorized into volume-, protocol-, and time-related features.

A critical challenge in IoT profiling lies in selecting a minimal yet informative set of features that accurately capture network behavior [9]. Existing approaches employ a variety of features, including size-related metrics (packet size, payload length, traffic rate, traffic volume), service-related indicators (protocol number, port number, DNS and Network Time Protocol (NTP) queries), time-related measures (flow duration, active/sleep duration), and statistical features (flow size, minimum, maximum), among others.

Notably, the in-depth analysis of DNS traffic generated by IoT devices has emerged as a valuable tool for device identification. In recent years, several research efforts have delved into this approach.

In [6], authors develop a lightweight DNS traffic monitoring system in edge networks to capture DNS traffic of IoT systems for behavioral analysis and modeling. This work starts with the study over time of DNS queries generated by IoT devices. They focus on two distinct levels of DNS names: (1) Fully Qualified Domain Name (FQDN) and (2) effective second-level domain (e2LD). Their system is based on a Bloom filter that monitors and detects emerging and novel FQDNs and e2LDs sent by IoT systems in order to detect anomalous behaviors.

The identification of the device type combined with vulnerability databases allows for spotting the vulnerability of the device, implying a privacy leak in IoT devices. Table 1 collects some works on device identification based on network traffic analysis. We specify (i) the dataset used (if it is public or if it was collected by the authors) and how many devices were analyzed; (ii) the number of features used and if DNS traffic is covered; and (iii) the best-performing machine learning technique, as well as the accuracy obtained.

Most of the works included in Table 1 extract features from network traffic, covering DNS traffic generated by devices.

Meidan et al. [10] used machine learning techniques to classify IoT devices based on tuples given by IP addresses and ports, extracted from the network traffic. The Random forest (RF) model obtained 99.28% accuracy in IoT device classification.

Miettinen et al. [11] proposed a system, called IoTSentinel, based on machine learning techniques to identify device types using 23 features at the packet level. The first set has 16 protocol-based features, and the remaining features include packet content, IP addresses, and ports. The classification system, based on the RF model, obtained 95% of accuracy in the identification of 17 devices and around 50% in the rest of the devices.

The authors of [12] presented a method for device type identification based on the behavioral fingerprinting performed. They used features extracted from network traffic at the packet level, 17 header features (based on the protocols used), and three payload features (entropy, TCP Window size, and payload length) to train machine learning models. A mean accuracy between 95% and 99% is obtained in the K-Nearest Neighbor (KNN), Decision Tree (DT), and Gradient Boosted Regression Tree (GBRT) models applied.

A distributed device fingerprinting technique (DEFT) to identify IoT devices is an approach proposed by Thangavelu et al. [13]. The traffic fingerprints of the devices are collected, and supervised models (RF, KNN, Gaussian, and Bernoulli Naive Bayes) are applied after dimensionality reduction to identify devices based on statistical characteristics extracted from application-layer protocols. Features of the DNS protocol considered are statistics of packet length and time between query and response, number of packets, number of queries, most queried domain name, and number of DNS errors. The RF classifier achieved the best performance, obtaining an accuracy of 98%, and Naive Bayes (NB) is the worst model, with 85% accuracy.

In [14], the authors applied RF and NB models to classify devices based on 12 extracted features, including domain names queried and DNS intervals regarding the DNS protocol. The accuracy obtained in the classification was 99.88%. Additionally, the authors studied the impact of the different features in the device identification.

Kumar et al. [16] evaluated different machine learning techniques using features of network traffic. The features used were divided into packet level (ports and packet lengths), flow level, and behavior level, including DNS Interval and DNS Queries. The following machine learning algorithms were evaluated: Neural Network (NN), Gaussian Naive Bayes (GNB), DT, RF, Support-Vector Machines (SVM), AdaBoost, XGBoost, Artificial Neural Network (ANN), Convolutional Neural Network (CNN), and Long short-term memory (LSTM). The best results obtained were 97.51% accuracy with CNN at the behavior level at the flow level, 80.67% accuracy with ANN, and at the packet level, 74.76% accuracy with CNN.

The approach presented in [17] used directional packet length sequences to identify the device based on machine learning techniques. The techniques applied were CNN and Multi-layer Perceptron (MLP), with 99% and 77% accuracy, respectively.

In [18], the authors presented IoTDevID, a method to identify devices eliminating redundant features based on feature selection and the genetic algorithm. In the classification, the Gradient Boosting (GB), DT, NB, KNN, RF, and SVM models are compared. The classification method relies on features extracted at the packet level, including features related to DNS protocol header fields.

AutoIoT [19] is a semi-supervised learning method used to distinguish between IoT and non-IoT devices and classify IoT devices with 95.82–99.96% accuracy. The authors used CNN to reduce the dimensionality of the extracted features. EvoIoT [21] also identifies IoT and non-IoT devices. The proposed model based on the RF technique is evaluated in different groups, in which the devices studied are grouped. The range of classification accuracy for all devices spans from 97% to 99%.

IoTTFID [23] is an incremental device-identification method based on device fingerprints. The average accuracy obtained for UNSW and Yourthings datasets is 98.19%. Characteristics of the application layer are analyzed, including features related to DNS protocol header fields and the size of responses.

Zhao et al. [24] performed efficient IoT device identification based on time series analysis and Logistic Regression (LR) considering packet lengths with directions with an accuracy of 99.81%. This study only considers two characteristics to define the devices pattern, and DNS traffic is not considered in the analysis conducted.

The only paper listed in Table 2 that uses only DNS traffic as a feature to characterize IoT devices is [15]. IoTFinder [15] is a machine-learning-based identification system that relies on DNS fingerprints of IoT devices. The authors collected passive DNS data from a wide range of IoT devices, although the collected data are not publicly available, and developed a system based on machine learning. IoTFinder achieves high accuracy in detecting various IoT devices based on the traffic generated within a local network or a network hosted behind a NAT.

In this paper, we focus on DNS traffic generated by different devices. We analyze the DNS traffic found and study the impact on the identification of the analyzed devices. The objective is to evaluate the capability of identification of an IoT device in an attack based on the information provided by the generated DNS traffic.

## 3. DNS Traffic Analysis

IoT devices generate DNS traffic mainly to communicate with Internet servers. In this section, we examine the different characteristics extracted from DNS traffic.

The analysis is focused (i) on the distinction between IoT and non-IoT devices based on the DNS traffic behavior pattern and (ii) the identification of different IoT devices.

### 3.1. Dataset

Some datasets containing IoT network traffic are available for the research community [26]. Some datasets used in the literature are summarized in Table 2. For comparison, the number of devices contained, the type of traffic captured, the duration of the traffic captured, and the availability of the dataset are shown.

In this study, we use the dataset collected by the University of New South Wales, abbreviated as UNSW [14]. The authors captured the traffic by building a testbed emulating a typical smart home environment. The traffic was captured over 26 weeks, although only a limited period is available for research.

This public trace contains network traffic from more than 30 devices, including IoT and non-IoT devices. These are devices ranging from appliances to health monitors, but also non-IoT devices such as computers, mobile phones, and tablets. Table 3 summarizes information about IoT devices categories. We analyze 27 IoT devices divided into the following categories: appliances, cameras, controllers, energy management, and health-monitors, and 7 non-IoT devices, devices involving user interaction or control, including computers (laptop, Macbook), mobile phones (Android phone, Android phone 2 and iPhone) and tablets (Samsung Galaxy Tab).

The overall duration of the dataset is approximately 60 days. The dataset contains both raw traffic in PCAP files and processed traffic in CSV files. We have used the raw (PCAP) traffic for our study. There are available daily network traces, and the size of the daily logs varies between 61 MB and 2 GB, with an average of 365 MB.

### 3.2. DNS Features Extraction

We process the raw traffic to obtain the information concerning the DNS queries made by the different devices. The network trace is not labeled, but we can identify the traffic generated by each device using the MAC address provided by the authors.

**Figure 1:**
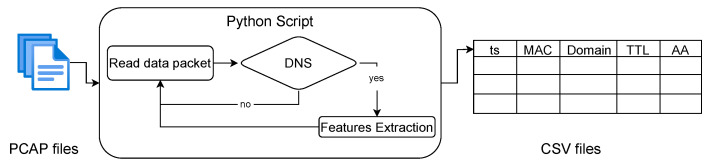
Processing network traffic data and extracting DNS features.

We read the data packets from the PCAP file and process the information obtained in each packet. We analyze the protocols present; if the packet contains the application layer, specifically, DNS, we analyze the content of the packet. The processing followed to extract the information from the devices at the DNS level is illustrated in Figure 1.

We filter the packets by port and packets containing a DNS layer. After the filtering is performed, we extract the DNS data features. The features extracted are as follows:

- Timestamp: The timestamp of the DNS packet.
- MAC Address: This address permits the identification of the device.
- DNS Domain: The domain name that has been queried for resolution.
- Resource Record (RR) type: The data record that identifies the type of RR that has been requested.
- Time To Live (TTL): The lifetime, in seconds, that the record remains in the cache.
- Authoritative Answer (AA): The flag that indicates if the response was sent by an authorized server for the queried domain name.

We obtain the features of the daily DNS traffic flow and, in addition, based on the MAC address, we extract the corresponding device. DNS traffic will differ in characteristics depending on the behavior of the flow pattern. In the following subsection, we analyze the traffic pattern of IoT and non-IoT devices based on the features extracted.

### 3.3. Domains Names

**Figure 2:**
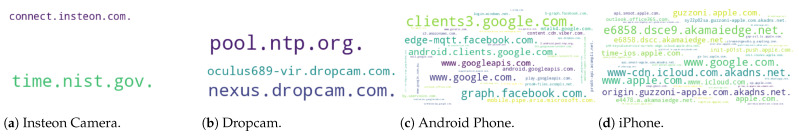
Word Cloud of domain names in devices.

Firstly, we analyzed the number of domains queried by IoT devices and compared them with non-IoT devices. It is important to analyze whether the domains consulted are significant. If the domains are unique, information about the device connected to the network can be revealed. As we can see in Figure 2, the domains are very significant. For example, the Insteon Camera only queries two domains, and one of them is connect.insteon.com, the manufacturer of the device is well-known due to the information provided. These domains could contribute to the identification of the different devices present in our environment, for example, in our home.

**Figure 3:**
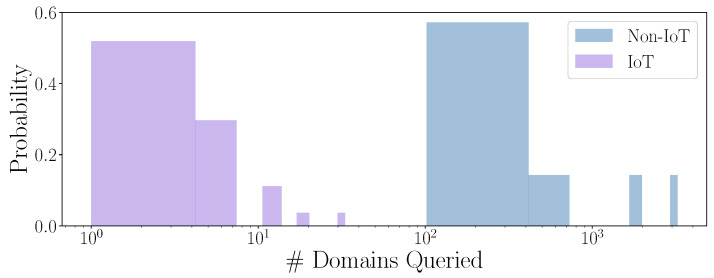
Number of different domains queried in IoT and non-IoT devices.

Note that IoT devices query fewer domains, and these domains are very significant. Next, we analyze the number of queried domains. In Figure 3, the distribution can be seen followed by both types of devices; note that the axis is in the logarithmic scale. IoT devices query very few domains because they can be used in a very specific context. The unique domains are concentrated in values of less than ten; specifically, an average of 6.2 domains are queried. However, in non-IoT devices, the user launches many different applications that query different domains with their own number of domains, thus leading to more diversity in the number of domains queried by non-IoT devices. In this case, these devices query an average of 903.7 domains, which amounts to almost 150 times more than IoT devices.

We want to study if the queried domains provide sufficient information to identify the devices connected to the network.

### 3.4. Machine Learning Classification

Machine learning techniques are applied to identify IoT devices based on the queried domains. The objective is to identify IoT and non-IoT devices considering only the domain names queried.

We consider the following machine learning techniques according to the related works [27,28,29]: DT, LR, NB, and RF. We randomly divide the data into two sets according to the following 80–20% split. The first set we use for classifier training and the second set as a test set to evaluate the different models and obtain the performance metrics.

The input variable to the classification models is a bag of domain names because the domain name is a categorical variable. We convert the categorical variable into numeric values following a binary encoding. We create binary columns for each domain queried. The resulting bag of domain names has the number of different domain queries as columns, and the number of rows corresponds to the number of queries performed by all devices.

**Figure 4:**
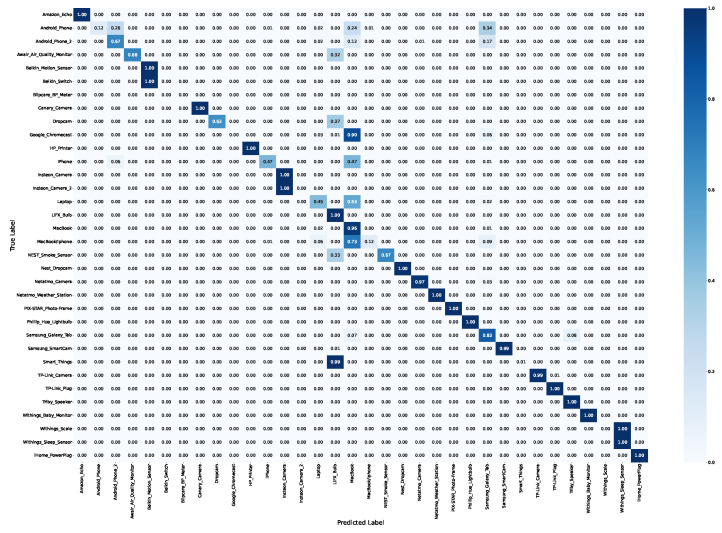
Confusion matrix of the model.

The results obtained in the RF model are collected in Figure 4, depicting the confusion matrix obtained in the classification. The overall accuracy obtained is 92.2% for the RF.

The confusion matrix obtained is a sparse matrix; by exploiting the matrix sparsity, misclassified samples can be further analyzed. The first case of misclassification is in the Belkin Motion Sensor and Belkin Switch devices; the second one is 100% wrongly classified as the first one, which is because both devices are developed by the same manufacturer. The Insteon cameras are another example of this hypothesis. The same results are obtained for the two devices developed by Withings. This implies that it is feasible to identify the manufacturer.

The sparsity observed in the confusion matrix further implies inherent limitations in distinguishing certain types of devices. Through careful analysis of results, general-purpose devices, such as the Macbook or Samsung Galaxy Tab, can be identified. One device is the iPhone, which achieves less than 50% of correct classification and is misclassified as a MacBook.

Table 4 shows the accuracy obtained in the classification. It shows that the domain names provide enough information to correctly identify the devices connected to the network.

We demonstrate that it is feasible to identify specific IoT devices with more than 92% accuracy based on the behavior of DNS traffic on the network, specifically, the domain names queried.

#### Comparison with Other Works

We made a comparison with the results obtained in other works in order to evaluate the previous classification and verify that the information provided by the domain names is sufficient. In [14], the authors present an architecture with different layers for IoT device classification. In the first layer, one feature is the bag of domain names, and taking into account only this variable, a precision of 79.48% is obtained using an NB classifier. Consequently, the results obtained are similar.

Additionally, we assess the results obtained in comparison with other studies mentioned above, particularly those employing the same dataset and machine learning methods based on DNS traffic. This comparison is presented in Table 5.

A detailed analysis of the DNS traffic generated will be provided in the following section, relating the domains consulted and the number of queries made.

## 4. Deep DNS Analysis

To provide insights into the presence of the DNS protocol in IoT devices, we analyzed the behavior pattern of queries performed and domains queried by each device.

### 4.1. Domains Names

**Figure 5:**
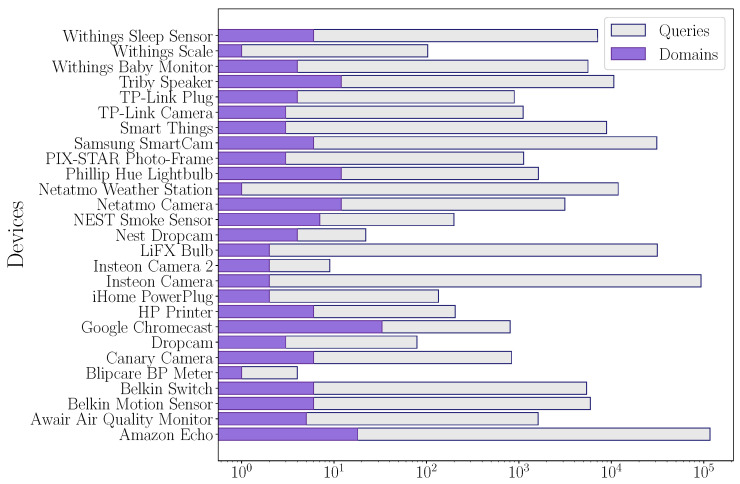
Number of different domains queried and queries by each device.

The number of queries made and the domains queried are important, as these will influence the information revealed and the likelihood that attackers will be able to extract that information. The number of queries performed and the number of domains queried are illustrated in Figure 5.

In this context, some IoT devices, despite consulting few domains, consult them repeatedly, in the order of 100,000 queries made by Amazon Echo and Insteon Camera. In addition, few domains are queried repeatedly, which reveals information about the device, leaving it exposed to future attacks that rely on device identification.

At first sight, one might expect that the number of domains consulted is directly related to the number of queries made, but it is not necessarily correlated. A device that queries few domains may repeat queries frequently; in the graph, this behavior can be observed in the Insteon Camera device.

Table 6 provides information about domains queried in detail. Also, the most frequently consulted domain and its occurrence as a percentage are shown.

In most devices, the most consulted domain is a very characteristic domain because it reveals information about the manufacturer of the device, or indeed, of the device. An example of this type of device is the Withings Baby Monitor device, and the most queried domain is babyws.withings.net.

The relation between the queried domains with the pattern of the behavior of the queries made would allow the attacker to extract information about the device. Next, we analyze the pattern of flow-related DNS queries.

### 4.2. Behavior Pattern

Depending on the DNS behavior, it would be possible to know when the device is being used because the DNS traffic generated by each device is different. To understand the behavior of the different devices, we analyzed the number of days during which the devices perform queries.

**Figure 6:**
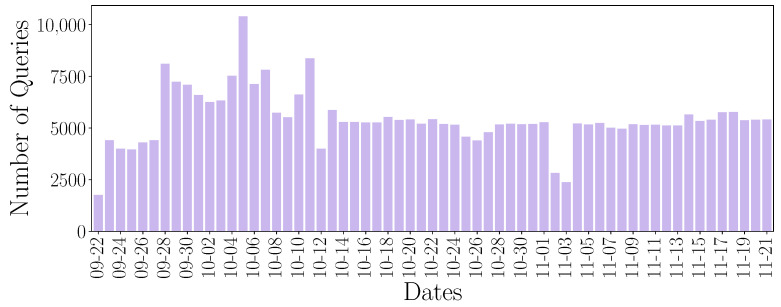
Number of queries generated per day by all devices.

The number of daily queries is illustrated in Figure 6. A fluctuation in the number of daily queries can be observed. This fluctuation may be due to the use of the different devices and their behavior pattern.

**Figure 7:**
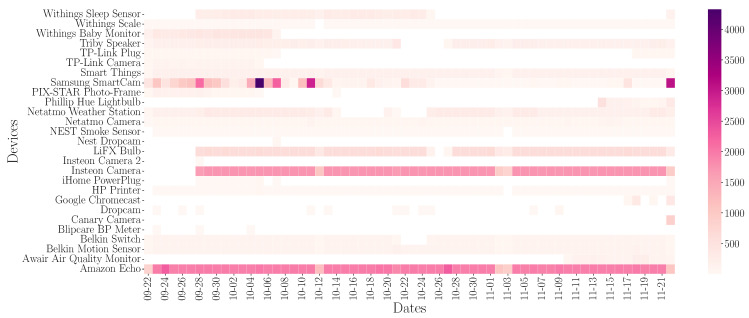
Number of daily queries generated by each device.

Figure 7 shows the distribution of queries made by the devices each day. Different behaviors can be highlighted. There are devices that perform queries every day, or even perform the same number of queries every day, presenting a recurrent behavior. This is the behavior presented by Amazon Echo. Other devices present the same DNS traffic flow, although the daily pattern is not presented every available day. And finally, we find the devices that do not present a periodic behavior, as would be the case of the Samsung SmartCam.

Different behaviors can be observed in the flow pattern of DNS queries: (a) queries performed periodically, (b) queries performed when the device is turned on, and (c) queries performed when the user interacts with the device.

In addition, the number of daily queries is shown. The purple color refers to the lower number of queries performed, while the yellow color reflects a higher number of queries performed. It can be seen that the Samsung SmartCam device presents a greater number of queries than the rest of the devices. This volume corresponds to the increase observed in Figure 6 around 5 October.

**Figure 8:**
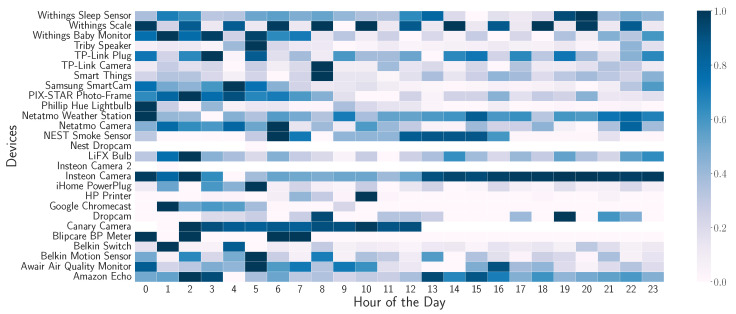
Pattern of behavior of the queries made by each device performed per day in a one-hour interval.

If we disaggregate the queries made on an hourly basis, we can observe the different behaviors in Figure 8. To obtain this distribution, we have grouped the queries made in an hour, and the average of all days is generated. By doing this, the hourly pattern of DNS queries on the different devices is obtained. The number of queries is normalized to observe the maximum and minimum relative to each device.

Each device has a different hourly pattern. Although a higher volume of DNS queries is observed during the early hours compared to the rest of the day. Other devices show a periodic pattern throughout the day, such as Awair Air Quality Monitor.

A time-related feature at the flow level is studied below after analyzing the queries performed individually at the packet level.

### 4.3. DNS Interval

The DNS interval is the time that elapses between two consecutive DNS queries made by a device. The elapsed times depend on the device configuration and the DNS cache implemented. DNS intervals provide insights into the behavioral pattern of the device.

**Figure 9:**
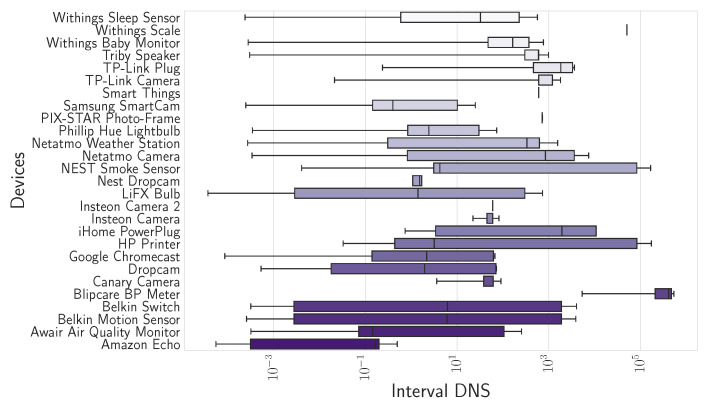
Range of DNS interval values determined, time elapsed between one query and the succeeding query (with logarithmic scale on the *X*-axis).

The difference between two queries for each device is calculated to analyze how often the query is repeated. The distribution of DNS intervals obtained can be seen in Figure 9. The X-axis scale in the figure is on a logarithmic scale to appreciate the distributions followed by the devices that perform queries more frequently, and therefore, its distribution is concentrated in low values. This is the case of Amazon Echo or Light Bulbs LiFX Smart Bulb. In addition, if the devices have focused DNS intervals in a narrow span, it implies that queries have been generated periodically because the same time elapses between one query and the next. This is the case of Insteon Camera.

The analysis conducted has revealed an unexpectedly high volume of queries, and IoT devices perform many queries to few domains. Although this may be due to different reasons, we will now evaluate the indispensability of the queries performed. Consequently, the reason for the queries performed will be deduced. In the next section, we will analyze the TTL of the queries made to evaluate the indispensability of the queries made.

## 5. Analysis of the High Volume of Queries

The volume of queries generated is conditioned by the implementation of the cache. DNS data remain in the cache for a specific time, the lifetime is determined by the TTL. However, if IoT devices have limited resources, the cache size could be imposed on a small size, causing repeated requests.

### 5.1. Time Space Analysis

**Figure 10:**
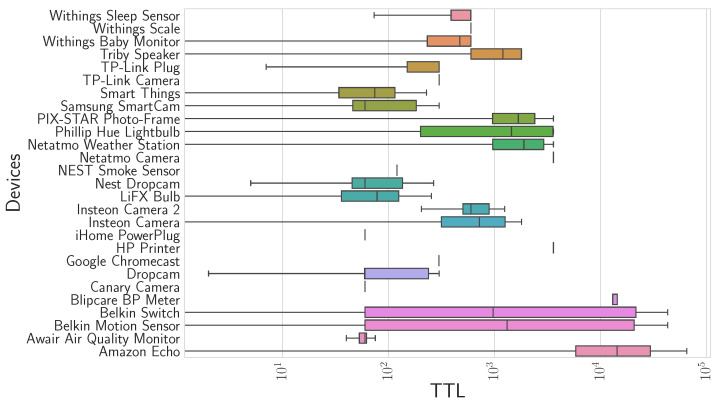
Range of TTL values obtained in the response section fields (with a logarithmic scale on the *X*-axis).

To examine the reason for the high volume of queries and to determine an imperative recurrence of requests, we studied the TTL size values obtained in the DNS packet field. The TTL determines how long a record will remain in the cache before it is deleted and needs to be queried again. We first analyze the TTL values obtained from the resource records of the examined DNS traffic to understand if the TTL influences the behavior pattern in the flow of DNS queries. The distribution of the TTL values can be observed in Figure 10. The histogram shows the distribution of all the devices.

A wide variability can be observed in most of the IoT devices. The average lifespan of responses received on the Belkin devices, Belkin Motion Sensor, and Belkin Switch, is higher than the rest.

High TTL values do not indicate anything; these values could indicate that queries may remain in the DNS cache for a long time. However, to obtain insight into the volume of queries generated, one can analyze the queries temporarily by studying the relationship between the TTL and the DNS interval.

**Figure 11:**
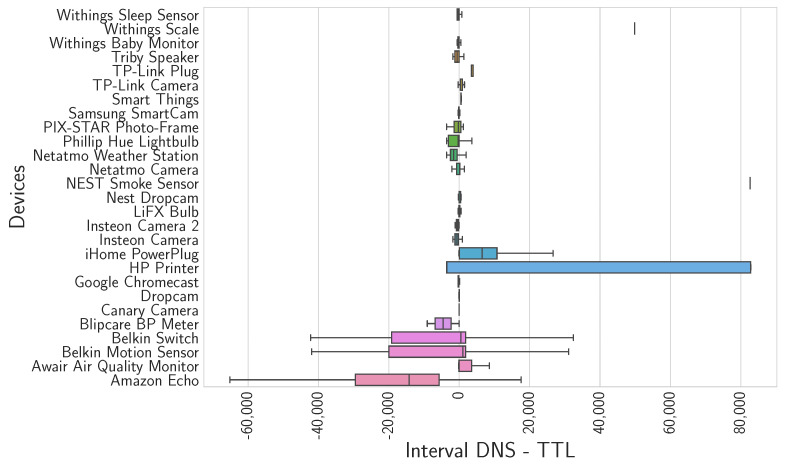
Values obtained from the difference between the DNS intervals and the TTL values of the corresponding response field, and values calculated for each domain.

Next, we analyze the TTL of the record and the timestamp of the next response received. Figure 11 shows the difference between the interval DNS obtained and the TTL value registered in the first response.

Getting negative values in the time difference means that the queries are repeated before the TTL expires. Therefore, we deduce that the devices do not cache as much as possible and explain the repetition of queries every short interval of time. The next question would be, assuming the devices had a DNS cache without limited size and could conserve the necessary resources, what insight the generated DNS traffic would provide.

### 5.2. Redundant Queries

The elapsed time between two queries compared to the TTL of the first query details the requirement of the queries performed. This analysis provides the queries performed before the TTL expires and, therefore, how many are performed without being required. We analyze the raw traffic packet by packet and filter the DNS traffic. The objective is to analyze what percentage of the queries performed are redundant and, therefore, how many queries are providing additional information about the devices.

We create a cache for each device, and in the cache, we store the queried domains and the timestamp when they will expire. The first step when a packet arrives is to remove the domains from the cache that have expired and check if the queried domain is in the cache, which would imply that the query is redundant. In addition, the cache is updated with all the RRs of the responses with the timestamp, obtained from the packet timestamp plus the TTL. As a result of the processing described above, we obtain data about the DNS queries performed and a flag that differentiates between redundant queries and queries according to the TTL received.

**Figure 12:**
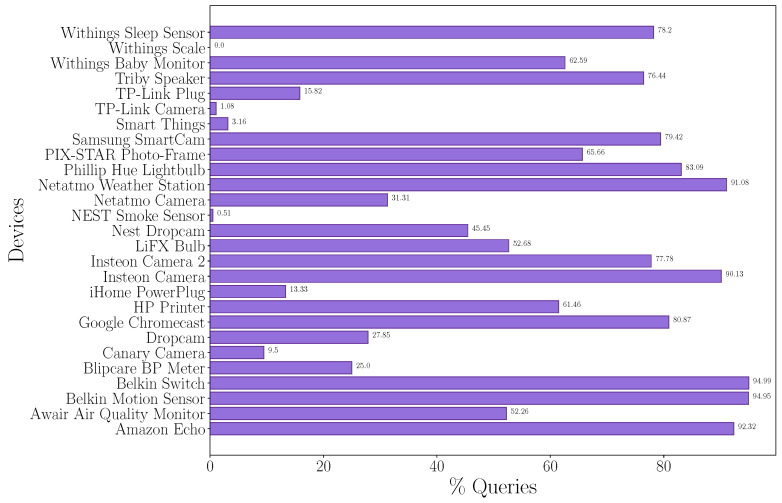
Percentage of redundant queries on each device compared to all queries analyzed.

The result obtained is shown in Figure 12, where the percentage of the queries performed that are redundant is illustrated. More than 90% of the queries generated by five devices (Amazon Echo, Belkin Wemo switch, Belkin Wemo Motion Sensor, Insteon Camera, and Netatmo Weather Station) are redundant. But analyzing the results, we discover that more than 50% of the queries generated by half of the devices were redundant. This means that the DNS traffic generated could be reduced by half if the devices had a large enough cache.

**Figure 13:**
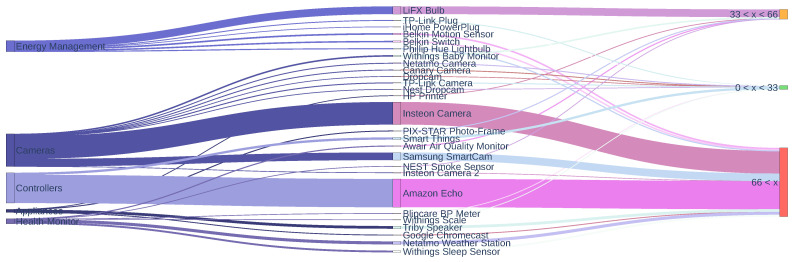
Sankey Diagram (IoT devices).

The flow diagram of the devices analyzed, as well as the device types and category, are shown in Figure 13. The last component of the diagram is the results obtained in the number of redundant queries in percentage.

This diagram shows the different behaviors of categorized DNS queries on different types of IoT devices. No pattern is observed in the volume of redundant queries by device type. However, one conclusion can be drawn, and that is that devices that generate a higher number of queries have a higher percentage of redundant queries. These devices are (i) Amazon Echo (controllers), (ii) Insteon Camera (cameras), (iii) LiFX Bulb (energy management), and (iv) Samsung SmartCam (cameras).

This analysis reveals a security problem in IoT devices. IoT devices present reduced caches, so more queries than necessary would be generated, resulting in a vulnerability because the information exchanged by the devices is increased; at the same time, attackers are more likely to listen to that information and identify the devices on the network more efficiently. IoT devices could be identified in real time based on their DNS queries. Depending on the DNS behavior, attackers could know at what time the device is being used; even in certain scenarios, we could infer whether users are at home or not from this information.

### 5.3. Evaluation of the Limitation-Redundant Queries

We apply the same techniques we have applied previously but remove redundant queries. We want to verify that the identification of the devices is less accurate if the traffic generated by the devices is reduced.

Table 7 shows the results obtained for all the machine learning techniques used compared to the previous classification. A decrease is noted in the accuracy obtained, and a a reduction of 10% was achieved in three techniques, and a reduction of up to 20% was achieved for the NB model.

If we quantify the accuracy obtained by the classification algorithms, we manage to reduce this accuracy by 10%.

### 5.4. Insights

We observed a high number of queries that would not be necessary if the TTL policy were complied with, and DNS queries are made when the TTL expires. The following conclusions can be drawn from the results obtained: (a) devices from the same manufacturer have queries to common domains; (b) IoT devices generate a high volume of queries to specific domains; and (c) the queried domains are limited. An IoT device is identifiable based on the queried domains, so it is important not to repeat queries excessively.

DNS traffic is sufficient to identify IoT devices connected to the network because the queried domains are characteristic. Nevertheless, restricting redundant queries would reduce the information provided and consequently render the identification of the device more difficult. Establishing new cache policies on IoT devices would increase privacy. In the next section, we evaluate different cache sizes to establish a suitable cache size.

## 6. Measure Proposed

The proposed measure is based on the simplification of the DNS cache following the implementation of a small and simplified cache of Android devices (https://android.googlesource.com/platform/bionic/+/master/libc/dns/resolv/res_cache.c (accessed on 27 February 2024)). This simplification buffers DNS responses for a specified time; this time is defined by the smallest TTL found in the response records. The goal is to reduce DNS traffic through a very simple design.

The objective of this study is analogous: a simple measure to reduce DNS traffic while maintaining device privacy. An excessively large cache is susceptible to a DNS spoofing attack, and attackers introduce malicious data into the DNS server cache [30].

In this section, we propose and evaluate different possible cache sizes. In addition, we evaluate the impact of this cache simplification on device identification.

### 6.1. Implementation

The implementation of the new cache policies is based on answer sizes, and the goal is to find the optimal size that enables the storage of a few answers without an excessive size, considering the privacy trade-off between having no cache and having a very large cache.

**Figure 14:**
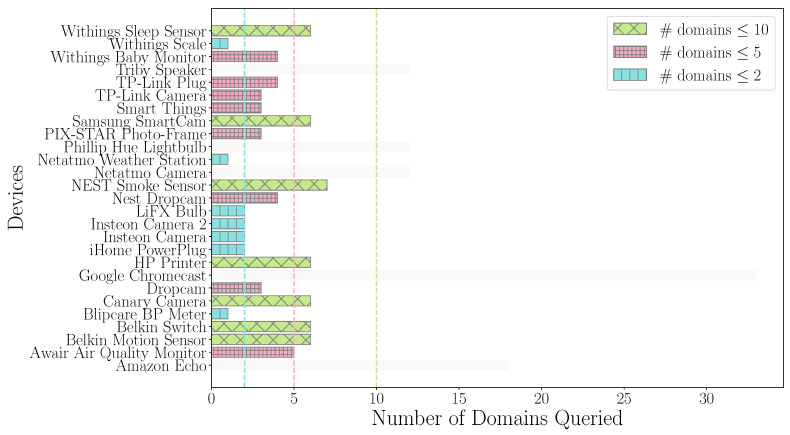
Number of domains consulted by each device. The blue color represents the devices that query fewer than two domains, the pink color represents fewer than five domains, and the green color represents fewer than ten domains.

First, to optimize the number of answers to be allocated in the cache size, we study the number of unique domains queried by each device. Figure 14 shows the number of unique domains queried by each device using three different levels. These settings will be the maximum number of records that can be stored in the cache.

Based on the above criteria, the sizes established for implementing the DNS cache and evaluating its impact are 2, 5, and 10. Additionally, a cache of unlimited size will be evaluated that is the worst possible scenario in terms of memory. This assumption would be the one requiring the largest capacity, which will be compared to the simplified cache implementation.

A cache is created where the obtained answers are stored; the limit size is the number of recorded answers. In addition, all the resources obtained in the response section are stored with the minimum TTL obtained in all the resources of the response section. First, expired cache entries are removed by comparing the current time with the lifetime of the cache entries. Secondly, the cache is checked to verify if the queried domain is found. If it is, the query would not be necessary and is marked as redundant, and if it is not found, it is added to the cache and updated with the new values.

Updating the cache involves two steps. The first step is checking the cache size, and if the cache has the maximum size set, the oldest response is removed and the current response is added. Then, the time to live is calculated as the sum of the packet timestamp and the minimum TTL found in the response section of the DNS packet.

The results obtained for the different sizes are presented below.

### 6.2. Required Queries

Based on the simplification of the cache, we obtain the number of DNS queries required with the different sizes established compared to the network traffic of the analyzed dataset. Table 8 shows the number of queries required as a percentage according to the different cache sizes established; the objective is to analyze the most appropriate cache size considering a compromise between privacy and usability.

Looking at the percentages obtained, from Cache^2^ to Cache^3^, there is a considerable decrease in some devices, including almost 50% for the Amazon Echo device. However, from the enlargement of Cache^3^ to Cache^4^, no such considerable fluctuations are observed. For this reason, we have selected this implementation for further evaluation.

One particular scenario is the Insteon Camera device, for which the results obtained are remarkable. The decrease when an infinite cache is implemented is outstanding. The reason lies in the fluctuation of the TTLs obtained in the TTL field resources in the response section; in the simplification, we used the minimum TTL. The cache entries are removed earlier, and therefore, the number of queries required is considerably higher.

On the other hand, devices that query very few different domains only need a very small cache size. Table 9 illustrates how the number of devices fluctuates relative to the percentage of traffic required across different cache sizes.

The first row indicates the number of devices that would generate less than 90% of the traffic considering the size of the cache. The number of devices decreases as the percentage of required traffic decreases. The variation in the number of devices is observed as the cache size increases. As previously mentioned, the difference between Cache^3^ and Cache^4^ is negligible, with only one device reducing the required traffic when the cache is expanded. As the trade-off between utility and privacy becomes relevant, it is imperative to consider that larger cache sizes entail higher costs. Therefore, we have established a cache size of five as the appropriate size, balancing considerations of privacy and usability.

Based on this implementation, the impact of the decrease in DNS traffic will be evaluated by applying machine learning techniques.

### 6.3. Evaluation

The proposed new cache policy will then be evaluated by setting the size to five resources. In this case, we want to analyze the impact of a possible attack when attackers are listening to a day’s traffic. We want to evaluate what information is provided during one day of usage.

The instances are the number of queries made to each domain in the interval of one hour. The objective is to obtain the devices connected to the network in the interval of one hour with the algorithm trained.

Data preprocessing is based on the grouping of queries made by each device on an hourly basis. The model is trained with the queried domain names; in the training matrix, the columns are the domains queried by the device, and the target is the device.

For the evaluation, we use 70% of the queries needed in this assumption to train the machine learning model. The RF model achieves 73% classification accuracy based on traffic captured on 22 November.

**Figure 15:**
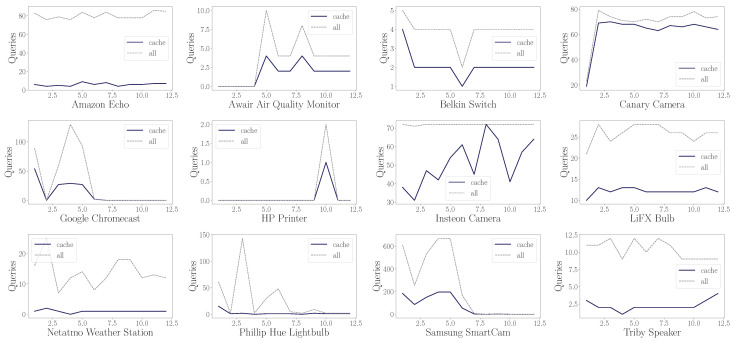
Difference between the number of queries generated by different devices during one day (22 November) on an hourly level.

One observation from the results obtained is that there is hardly any difference in DNS traffic over the hours. However, analyzing the number of queries of all traffic with respect to the number of queries after implementing the cache simplification results in a lower number of queries over the hours, as can be seen in Figure 15; however, at the hourly level, queries are still obtained. Accordingly, this is the behavior observed for the devices analyzed.

The advantages achieved with this proposed measure are (i) a reduction in generated DNS traffic, (ii) a reduced number of stored responses, and (iii) increased privacy because less information about the device is exposed.

## 7. Conclusions

The deployment of Internet of Things (IoT) devices is widespread in different environments, including homes. These devices bring automated services to homes that incorporate security and energy efficiency. However, homes become targets for cyberattacks because IoT devices can present certain vulnerabilities. IoT devices generate DNS traffic primarily for communication with Internet servers.

In this paper, we have demonstrated from real IoT traffic traces that DNS traffic provides sufficient information to identify IoT devices. Furthermore, IoT devices generate more DNS queries than necessary. Despite the often limited number of distinct domain names consulted and the long time to live (TTL) of response records allowing for caching and minimizing queries, the absence of a cache or the implementation of overly simplistic caching strategies in IoT devices results in a significantly higher DNS query volume than expected. This easily enables an observer of such traffic to discern the types of devices present in the network.

Implementing novel policies for the DNS cache in IoT devices increases user privacy while still preserving the trade-off between privacy and usability, because IoT devices have limited capabilities. We propose a simple measure in implementing a DNS cache simplification to store at most five responses with the minimum possible TTL. This implementation leads to a decrease in the DNS traffic generated, rendering the identification of an IoT device in a potential attack more difficult.

In future work, we will quantify the extent to which the utilization of caches, in conjunction with differential privacy mechanisms, can prevent an attacker from using DNS queries to identify devices, and we will assess the associated costs. In addition, we will evaluate the impact of the use of secure DNS protocols on device identification, as well as the enhancement of privacy.

## Figures and Tables

**Table 1:** Related work review with respect to the dataset, features extracted used in the classification, and techniques used. N.A.: Not Available.

Reference	Year	Collect Data	Public Dataset	# Devices		Extracted Features	DNS	ML	Accuracy
		Available		IoT	Non-IoT				
Meidan et al. [10]	2017	N.A.		9	4	4		RF	99%
Miettinen et al. [11]	2017	Aalto		27		23	✓	RF	85%
Bezawada et al. [12]	2018	N.A.		10		20	✓	GBRT	99%
Thangavelu et al. [13]	2018	N.A.		16		111	✓	RF	98%
Sivanathan et al. [14]	2018	UNSW		28		12	✓	NB + RF	99%
Perdisci et al. [15]	2020	IoTFinder		53			✓	developed system	100%
Kumar et al. [16]	2021		[14]	20		14	✓	CNN	97%
Liu et al. [17]	2022			18		2		CNN	99%
Kostas et al. [18]	2022		[11,14]	31	28	112	✓	RF	94%
Fan et al. [19]	2022		[14,20]	69			✓	CNN	99%
Fan et al. [21]	2022	N.A.	[14,22]	71		56		RF	99%
Hao et al. [23]	2023		[14,20]	20	30	50	✓	-	98%
Zhao et al. [24]	2023		[14,25]	19	28	2		LR	99%

**Table 2:** Comparison of different datasets used in related work.

Dataset	Year	Devices		Type			DNS	Duration	Availability
		Non-IoT	IoT	Idle	Interaction	Setup			
UNSW [14]	2018	3	28	✓	✓		✓	2 months (September 2016)	✓
IoTSentinel [11]	2018	0	31			✓	✓	20 times/device	✓
IoTFinder [15]	2020	0	53	✓	✓		✓	2 months (August 2019)	✓
YourThings [20]	2020	0	45	✓	✓		✓	13 days (March 2019)	✓

**Table 3:** List of IoT devices included in UNSW dataset [14] and analyzed in this study.

Appliances	Cameras	Controllers	Energy Management	Health-Monitor
Google Chromecast	Canary Camera	Amazon Echo	Belkin Motion Sensor	Awair Air Quality Monitor
HP Printer	Dropcam	Smart Things	Belkin Switch	Blipcare BP Meter
PIX-STAR Photo-Frame	Insteon Camera		iHome PowerPlug	NEST Smoke Sensor
Triby Speaker	Insteon Camera 2		LiFX Bulb	Netatmo Weather Station
	Nest Dropcam		Phillip Hue Lightbulb	Withings Scale
	Netatmo Camera		TP-Link Plug	Withings Sleep Sensor
	Samsung SmartCam			
	TP-Link Camera			
	Withings Baby Monitor			

**Table 4:** Accuracy obtained in classification.

	DT	LR	NB	RF
Accuracy	0.9221	0.9202	0.7993	0.9223

**Table 5:** Comparison with related work.

Reference	Method	Features	Accuracy
This work	RF	1	0.92
[14]	NB	1	0.79
[14]	NB + RF	12	0.99
[16]	CNN	6	0.97
[18]	RF	112	0.94

**Table 6:** Details on the queries generated by each device.

Device	Queries	Domains	% Frequent	Domain
Amazon Echo	116,760	18	30.43	www.example.com.
Awair Air Quality Monitor	1617	5	37.66	pool.ntp.org.
Belkin Motion Sensor	5398	6	48.13	d3gjecg2uu2eaq.cloudfront.net.
Belkin Switch	5927	6	48.8	d3gjecg2uu2eaq.cloudfront.net.
Blipcare BP Meter	4	1	100	tech.carematix.com.
Canary Camera	832	6	85.1	b.canaryis.com.
Dropcam	79	3	40.51	pool.ntp.org.
Google Chromecast	804	33	67.83	www.google.com.
HP Printer	205	6	63.41	chat.hpeprint.com.
iHome PowerPlug	135	2	68.89	api.evrythng.com.
Insteon Camera	93,251	2	83.33	time.nist.gov.
Insteon Camera 2	9	2	88.89	time.nist.gov.
LiFX Bulb	31,493	2	99.51	pool.ntp.org.
Nest Dropcam	198	7	34.34	nexus.dropcam.com.
NEST Smoke Sensor	22	4	27.27	frontdoor.nest.com.
Netatmo Camera	3143	12	70.89	apicom.netatmo.net.
Netatmo Weather Station	11,869	1	100	netcom.netatmo.net.
Phillip Hue Lightbulb	1624	12	62.61	dcp.cpp.philips.com.
PIX-STAR Photo-Frame	1120	3	98.49	api.pix-star.com.
Samsung SmartCam	31,101	6	55.91	smtp.gmail.com.
Smart Things	8887	3	99.29	pool.ntp.org.
TP-Link Camera	1110	3	97.84	aps1-relay.tplinkcloud.com.
TP-Link Plug	891	4	59.82	uk.pool.ntp.org.
Triby Speaker	10,654	12	94.34	sip.invoxia.com.
Withings Baby Monitor	5586	4	99.86	babyws.withings.net.
Withings Scale	103	1	100	scalews.withings.net.
Withings Sleep Sensor	7119	6	99.45	scalews.withings.net.

**Table 7:** Accuracy obtained in classification.

	DT	LR	NB	RF
All data	0.9221	0.9202	0.7993	0.9223
No redundant	0.8206	0.8150	0.5812	0.8207

**Table 8:** Percentage of the number of queries required according to the different cache sizes implemented. Cache^1^: Cache with only one entry; Cache^2^: Cache with two entries; Cache^3^: Cache with five entries; Cache^4^: Cache with ten entries; Cache^5^: Cache without any restrictions.

Devices	Cache^1^	Cache^2^	Cache^3^	Cache^4^	Cache^5^
Size	1	2	5	10	inf
	(Resource)	(min TTL)	(min TTL)	(min TTL)	(TTL)
Amazon Echo	53.04	52.84	8.22	8.16	8.03
Awair Air Quality Monitor	56.52	47.87	47.8	47.8	47.8
Belkin Motion Sensor	99.65	51.17	51.02	51.02	50.69
Belkin Switch	99.27	50.51	50.18	50.18	49.86
Blipcare BP Meter	75	75	75	75	75
Canary Camera	90.87	90.5	90.5	90.5	90.5
Dropcam	96.2	89.87	72.15	72.15	72.15
Google Chromecast	43.41	39.55	37.31	36.69	24.75
HP Printer	39.51	42.93	42.93	42.93	38.54
iHome PowerPlug	90.37	86.67	86.67	86.67	86.67
Insteon Camera	35.76	68.22	68.22	68.22	13.62
Insteon Camera 2	33.33	100	100	100	22.22
LiFX Bulb	47.12	47.09	47.09	47.09	47.09
Nest Dropcam	100	100	54.55	54.55	54.55
NEST Smoke Sensor	99.49	99.49	99.49	99.49	99.49
Netatmo Camera	76.52	83.49	82.41	79.19	70.09
Netatmo Weather Station	9.18	9.18	9.18	9.18	9.18
Phillip Hue Lightbulb	34.67	23.28	18.9	18.35	18.35
PIX-STAR Photo-Frame	36.16	35.45	35.36	35.36	35.36
Samsung SmartCam	59.99	36.09	36.04	36.04	24.49
Smart Things	96.57	96.65	96.65	96.65	96.55
TP-Link Camera	99.37	99.1	99.1	99.1	99.01
TP-Link Plug	84.29	84.18	84.18	84.18	84.18
Triby Speaker	25.84	24.68	24.27	24.23	24.21
Withings Baby Monitor	37.7	37.68	37.68	37.68	37.68
Withings Scale	100	100	100	100	100
Withings Sleep Sensor	22.12	21.89	21.81	21.81	21.81

**Table 9:** Number of devices in relation to the percentage of required traffic according to the different cache sizes implemented. Cache^1^: Cache with only one entry; Cache^2^: Cache with two entries; Cache^3^: Cache with five entries; Cache^4^: Cache with ten entries; Cache^5^: Cache without any restrictions.

Required Traffic	Cache^1^	Cache^2^	Cache^3^	Cache^4^	Cache^5^
<90%	17	20	21	21	22
<80%	16	16	18	19	20
<70%	14	15	16	16	17
<60%	14	14	15	15	17
<50%	11	11	12	12	15

## Data Availability

Data are contained within the article.

## Abbreviations

The following abbreviations are used in this manuscript:

ANN	Artificial Neural Network
CNN	Convolutional Neural Network
DNS	Domain Name Server
DT	Decision Tree
e2LD	effective second-level domain
FQDN	Fully Qualified Domain Name
GB	Gradient Boosting
GNB	Gaussian Naive Bayes
GBRT	Gradient Boosted Regression Trees
IoT	Internet of Things
KNN	K-Nearest Neighbors
LR	Logistic Regression
LSTM	Long short-term memory
MLP	Multi-layer Perceptron
NB	Naive Bayes
NN	Neural Network
NTP	Network Time Protocol
SVM	Support-Vector Machines
TTL	Time To Live
UDP	User Datagram Protocol
RF	Random Forest
